# A method to tune the shape of protein-encapsulated polymeric microspheres

**DOI:** 10.1038/srep12634

**Published:** 2015-07-30

**Authors:** Renato de Alteriis, Raffaele Vecchione, Chiara Attanasio, Maria De Gregorio, Massimiliano Porzio, Edmondo Battista, Paolo A. Netti

**Affiliations:** 1 Centro di Ricerca Interdipartimentale sui Biomateriali - CRIB, Università degli studi di Napoli Federico II, Napoli, 80125, Italy; 2 Center for Advanced Biomaterials for Health Care - IIT@CRIB Istituto Italiano di Tecnologia, Napoli, 80125, Italy; 3 Dipartimento di Medicina Veterinaria e Produzioni Animali, Napoli, 80137, Italy

## Abstract

Protein encapsulation technologies of polymeric microspheres currently in use have been optimized to effectively protect their “protein cargo” from inactivation occurring in biological environments, preserving its bioactivity during release up to several weeks. The scenario of protein delivery would greatly benefit by strategies enabling the production of non-spherical particles. Herein we report an easy and effective stamp-based method to produce poly-lactic-glycolic-acid (PLGA) microparticles encapsulating Vascular Endothelial Growth Factor (VEGF) of different shapes. We demonstrate that PLGA microspheres can be deformed at room temperature exploiting solvent/non-solvent plasticization in order to preserve the properties of the starting microspheres. This gentle method allows the production of shaped particles that provide a prolonged release of VEGF in active form, as verified by an angiogenic assay. The retention of the biological activity of an extremely labile molecule, i.e. VEGF, lets us hypothesize that a wide variety of drug and protein encapsulated polymeric microspheres can be processed using this method.

Protein-encapsulated polymeric microspheres have been proved effective in releasing even very labile bioactive moieties in a specific manner at pre-programmed rates^1^,^2^,^3^. These systems effectively protect their “protein cargo” from inactivation occurring in biological environments and preserve its bioactivity during the release process^4^. For instance, Vascular Endothelial Growth Factor (VEGF), a potent angiogenic molecule, has to be properly encapsulated to allow its effective release over time, since it is extremely sensitive to environmental inactivation and is otherwise non-usable. Indeed, poly-lactic-glycolic-acid (PLGA) microspheres with elaborate architecture and formulation, loaded with VEGF complexed with heparin (Hp) and provided with a protective layer of bovine serum albumin (BSA), prolong the half-life of VEGF allowing its release in active form up to several weeks^5^,^6^. In addition, protein release profiles can be engineered by tuning microsphere size and microstructure with well-established protocols^7^,^8^,^9^.

Although current protein encapsulation technologies have been optimized for the production of microspheres, the scenario of protein delivery would be greatly expanded by strategies that enable the production of particles with more complex shapes than merely spherical. Indeed, there is a growing body of evidence supporting the importance of the role of the shape of polymeric microparticles, especially in the fields of drug delivery and tissue engineering^10^,^11^. In terms of drug delivery, particle shape affects many *in vivo* performances, such as transport, targeting and internalization^12^,^13^,^14^,^15^,^16^. Furthermore, needle-shaped particles, i.e. microneedles, of hundreds of microns in size provide an effective tool for transdermal drug delivery^17^,^18^,^19^. In the field of tissue engineering, particle shape plays a key role in the so called bottom-up approach, wherein shaped microparticles mimic the microenvironment of specific tissues and are used as building blocks for their construction^20^,^21^,^22^,^23^.

Various *ab initio* methods to produce microparticles with complex shapes have been described^12^,^24^,^25^,^26^,^27^,^28^ but, considering the expertise already available for spherical particle technology, the use of microspheres as a starting material to obtain microparticles with different shapes is particularly attractive. Mitragotri *et al*. proposed to deform previously fabricated microspheres embedded in a polymeric matrix into non-spherical geometries^29^. In particular, microparticles with a wide variety of shapes can be obtained liquefying microspheres, by means of heating or immersion in solvent, and stretching of the polymer matrix. However, the liquefaction of microspheres might affect the biological activity of labile embedded biomolecules and their effective release. In particular, this deformation method might alter the microstructure of the microspheres and the distribution of the protein cargo and its protective layer, thus jeopardizing the beneficial properties of the encapsulation strategies achieved for spherical particles.

Driven by the willingness to better exploit the advantages related to the use of microspheres as a starting material, and considering the possible effects of liquefaction, the aim of this study was to develop a novel stamp-based technique to produce shaped and isolated microparticles by deforming previously fabricated microspheres under gentle process conditions, i.e. at room temperature using a solvent/non-solvent vapor mixture, and to assess the release of VEGF in active form from the shaped microparticles over time.

## Results

### Shaping process

**Figure 1:**
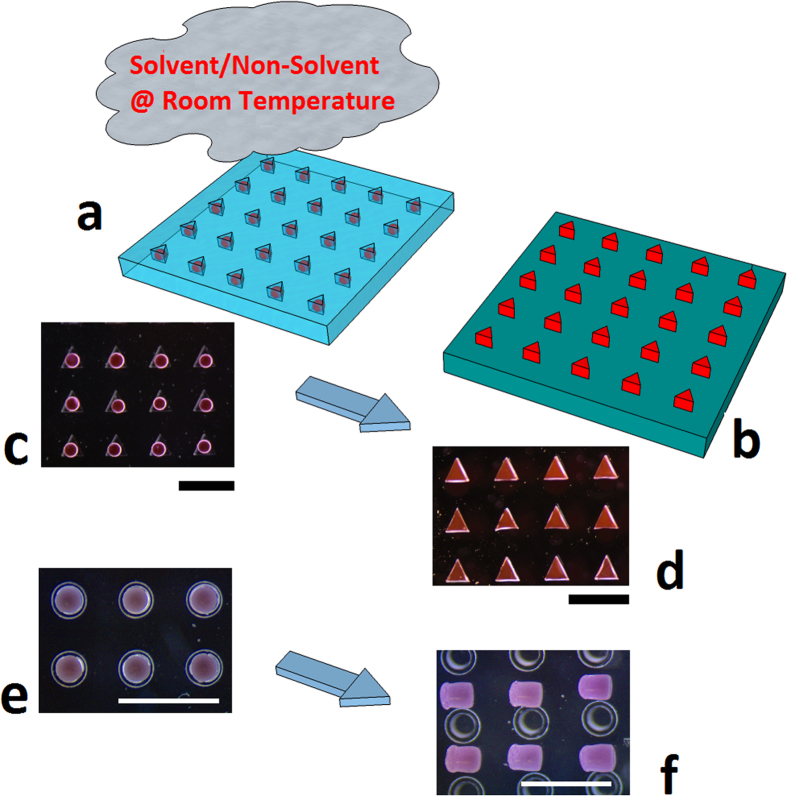
(**a**,**b**) PLGA microspheres loaded with NR, inside triangular prism-shaped cavities of a PDMS mold, exposed to a solvent/non-solvent (DMC/EtOH) vapor mixture at room temperature and released on a substrate; Stereomicroscope images of the starting and deformed microspheres with triangular (**c**,**d**) and cylindrical (**e**,**f**) shape. The cylindrically shaped cavities have an enlarged top portion. PLGA, *polylacticglycolic acid*; NR, *nile red*; PDMS, *polydimethylsiloxane*; DMC, *dimethylcarbonate*; EtOH, *Ethanol*. Scale bars 750 μm.

The main steps of the process to produce shaped microparticles starting from previously fabricated spherical particles are schematically shown in Fig. 1. As reported in the Methods section, Nile Red (NR)-loaded PLGA microspheres were placed inside the cavities of an elastomeric mold, exposed to a solvent/non-solvent vapor mixture, i.e. dimethylcarbonate (DMC) and Ethanol (EtOH), respectively, and demolded (Fig. 1a,b). In particular, NR-loaded microspheres were deformed into three different shapes, namely triangular prism (Fig. 1c,d), parallelepiped with a pyramid on top, and cylinder (Fig. 1e,f).

For further experiments, VEGF-loaded microspheres were shaped only into cylindrical particles, since the mold designed with cylindrical cavities allowed to produce an amount of particle suitable for the following VEGF bioactivity analysis. In particular, the enlarged top portion of the mold facilitated the filling of the lower portion, which was the actual molding cavity with cylindrical shape.

**Figure 2:**
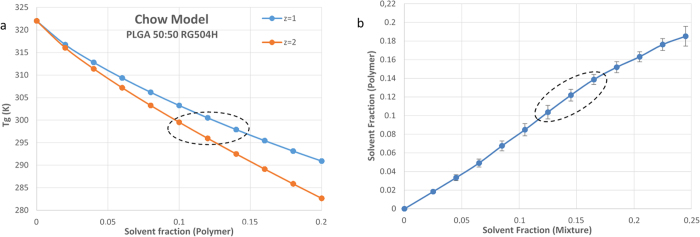
(**a**) Chow Model for the stock PLGA 50:50; (**b**) Comparison of the mass fraction of solvent in each solution with the corresponding solvent mass fraction within the PLGA film with related standard error, as evaluated by QCM. PLGA, *polylacticglycolic acid*; QCM, *Quartz crystal microbalance*.

Suitable solvent/non-solvent liquid mixtures to be vaporized onto microspheres were determined taking into account the Chow model^30^ (see Supplementary Information) for stock PLGA and quartz crystal microbalance (QCM) measurements on a thin PLGA film. To apply the Chow model, the glass transition temperature of the pure polymer (Tg_0_) and the change in specific heat (ΔCp) of the polymer associated with its glass transition temperature were determined *via* differential scanning calorimetry (DSC). DSC thermograms for stock PLGA revealed a Tg_0_ of 48.87 °C, in agreement with the manufacturer’s specification and ΔCp = 0.595 J (g K)^−1^. According to the Chow equation, the required range of DMC mass fraction *w* in the polymer required to lower the Tg_0_ of the stock PLGA to room temperature was approximatively 0.1–0.14 (Fig. 2a).

Comparing the mass fraction of solvent in each solution with the corresponding solvent mass fraction within the polymer film, as evaluated by QCM (Fig. 2b), and taking into account the Chow model, we could determine a narrow range of DMC mass fractions within solution (about 0.12−0.17) that could effectively plasticize the PLGA film at room temperature, while avoiding its dissolution. Furthermore, the increase in dissipation registered by the QCM instrument at such range (see Supplementary Information, Figure S1) gave a qualitative indication of the variations in the elastic modulus of the polymer^31^.

Although there cannot be an exact correspondence between the sorption behavior of the film and microspheres, the results from the Chow model and QCM measurements were useful to find a starting range of solutions. The best DMC mass fraction within solution to plasticize microspheres at room temperature was experimentally determined to be about 0.145. We found that exposure time was dependent on microsphere porosity. Indeed, while 7 min were required to deform microspheres with a pore size of 2–5 μm, 1 min was sufficient for microspheres with a pore size of one order of magnitude larger. Such pore sizes corresponded to microspheres produced with the Micropore® system and the VEGF-loaded microspheres, respectively (see Supplementary Information, Figure S2).

### Particle characterization and molecule distribution

**Figure 3:**
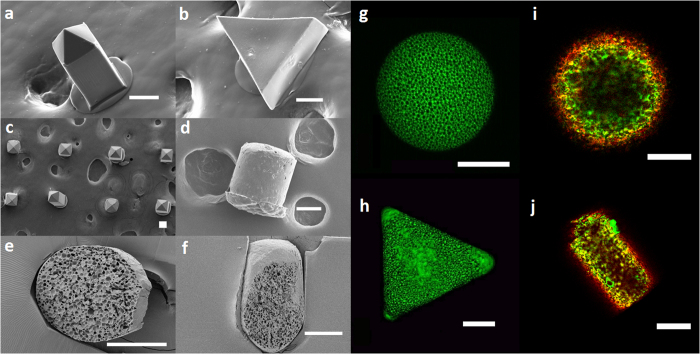
SEM images of deformed microspheres loaded with NR (**a**–**c**), and of a deformed microsphere loaded with VEGF/Hp/BSA (**d**); SEM images of a sectioned microsphere (**e**), and of a sectioned deformed microsphere (**f**). Confocal microscope z-stack maximum projection of a microsphere (**g**), and of a deformed microsphere (**h**), both loaded with NR. Confocal microscope images of a microsphere (**i**) and of a deformed microsphere (**j**), both loaded with labeled BSA, red, and Hp, green. The microstructure (**e**–**h**) and the distribution of BSA and Hp (**i**,**j**) in the shaped microparticles are very similar to those of the starting microspheres. SEM, *Scanning electron microscope*, NR, *Nile Red*, VEGF, *Vascular Endothelial Growth Factor*; Hp, *heparin*, BSA, *bovine serum albumin*. Scale bars 75 μm.

Scanning electron microscope (SEM) images of the resulting microparticles showed that deformation was achieved (Fig. 3a–d). In addition, SEM images of particle sections show that starting and deformed microspheres have a similar porosity (Fig. 3e,f).

Likewise, confocal microscope analysis of a NR-loaded microsphere and triangular microparticle (Fig. 3g,h) shows that -although a relatively higher concentration of NR can be observed at the corners- the starting and deformed microspheres had a similar porosity. Moreover, also the distribution of biomolecules in the starting and deformed microspheres loaded with labeled BSA and Hp was comparable (Fig. 3i,j).

### VEGF release and bioactivity

**Figure 4:**
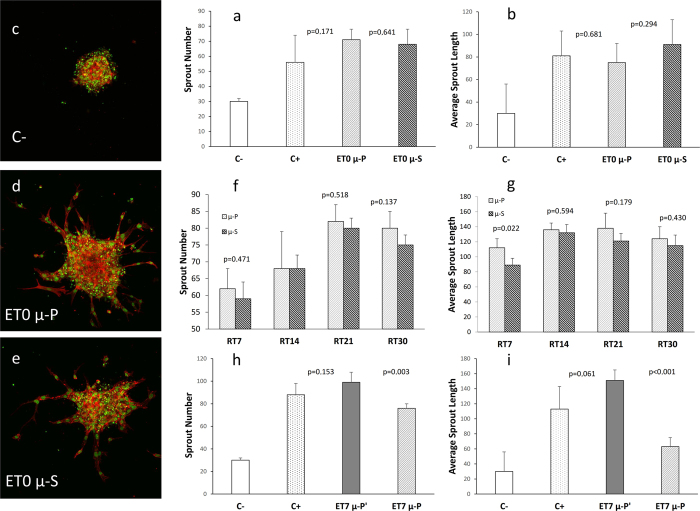
Proangiogenic activity of VEGF extracted or released from PLGA microspheres (μ-S) and deformed microspheres (μ-P), evaluated on HUVEC cells and measured as sprout number and average sprout length (μm). (**a**,**b**) sprout number and average sprout length, respectively, of the VEGF extracted from μ-S and from μ-P at baseline (ET0). ET0 μ-P and ET0 μ-S present equal VEGF activity. Confocal images confirming a lower angiogenic response in C- (**c**), and the evidence of sprouting in both ET0 μ-P (**d**) and ET0 μ-S (**e**). (**f**,**g**) sprout number and average sprout length, respectively, of the VEGF released from μ-S and from μ-P, at 4 different time points (RT7, RT14, RT21, RT30). When evaluating the sprout number, μ-S and μ-P present a similar VEGF activity at each time. When evaluating the sprout length, after 7 days of incubation (RT7) μ-P present a significantly higher VEGF activity. (**h**,**i**) sprout number and average sprout length, respectively, of the VEGF extracted from deformed microspheres after 7 days (ET7) of incubation, wherein μ-P’ were deformed with a longer exposure time to solvent as compared to μ-P. ET7 μ-P’ presents a higher residual VEGF activity, due to a lower release, as compared to that of ET7 μ-P. P values <0.05 were considered statistically significant. P values compared to C- were always <0.01. C−, *negative control*; C+, *positive control*; VEGF, *Vascular Endothelial Growth Factor*; HUVEC*, Human Umbilical Vein Endothelial Cells;* PLGA, *poly-lactic-glycolic acid*.

Results of the proangiogenic activity (evaluated as sprout number and average sprout length) of VEGF extracted or released from starting and deformed microspheres are summarized in Fig. 4. Interestingly, at baseline, i.e. after deformation, the proangiogenic activity of VEGF within the deformed microspheres was not statistically different from that of VEGF within the starting microspheres (Fig. 4a,b).

Confocal images confirmed a lower angiogenic response in the negative control (Fig. 4c), and the evidence of sprouting in both starting and deformed microspheres (Fig. 4d,e).

Figure 4f,g show the sprout number and average sprout length, respectively, of the VEGF released from starting and from deformed microspheres, at 4 different time-points. At each time, the activity of VEGF released in the cell culture medium was similar for both samples when evaluated as sprout number (Fig. 4f). This finding denoted that both samples released comparable quantities of VEGF in the cell culture medium at each time, thus confirming that starting and deformed microspheres have a similar porosity. Interestingly, we observed a slight but significantly higher sprout length from deformed microspheres compared to the starting microspheres, after 7 days (Fig. 4g), possibly due to a higher release caused by the difference in shape. The residual VEGF counterpart still entrapped within starting and deformed microspheres after incubation was also analyzed. In particular, samples showed a decreasing trend in the VEGF activity over time, thus confirming an effective VEGF release (see Supplementary Information, Figure S3). Figure 4h,i show data relative to the comparison study between microspheres deformed with the same plasticizing solution, but for different exposure times. The activity of residual VEGF embedded in the deformed microspheres was higher when exposed for a longer rather than a shorter time to the solvent/non solvent mixture due to a lower release, denoting that an excessive plasticization leads to closure of the pores of the deformed microspheres.

## Discussion

**Figure 5:**
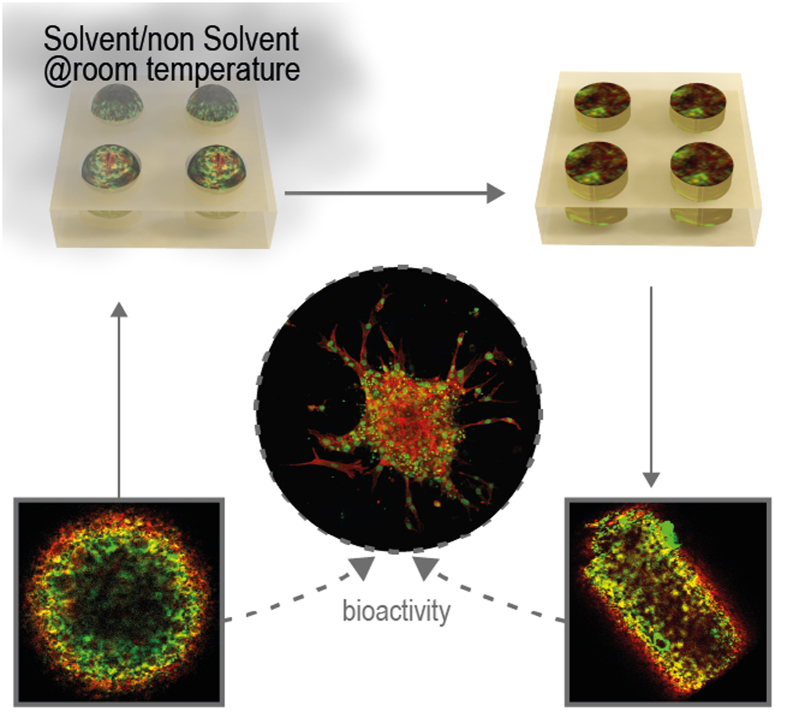
Schematic representation of the deformation process under gentle conditions. Working at room temperature with solvent vapors instead of liquid solutions enabled to preserve the properties of the starting microspheres, such as microstructure and embedded biomolecule distribution, and to produce shaped microparticles that can release active VEGF. VEGF, *Vascular Endothelial Growth Factor*.

We propose a new and effective method to produce non-spherical polymeric particles starting from previously fabricated PLGA microspheres loaded with VEGF/Hp/BSA exploiting solvent/non-solvent plasticization at room temperature using a vapor mixture. In particular, we used a vaporized mixture of DMC -a non-toxic solvent for PLGA- and EtOH -known to be a non-solvent for such polymer. Working at room temperature with solvent vapors instead of liquid solutions enabled us to enhance the gentleness of the process, thus preserving the properties of the starting microspheres, such as microstructure and embedded biomolecule distribution, and producing shaped microparticles that can release active VEGF over time. Our main results are summarized in Fig. 5.

It is known that an increase in macromolecule mobility is necessary to achieve plastic deformation^32^. In the case of amorphous polymers like PLGA, such deformation is typically obtained by heating them up to some tens of degrees above their glass transition temperature (Tg), which for commercial PLGA 50:50 is comprised between 46 and 50 °C. However, since many drug molecules are thermo-labile, such relatively high temperatures should be avoided in order to retain the biological activity of the embedded molecules as well as the particle microstructure.

In view of the above, we exploited the phenomenon of solvent plasticization, that is the depression of Tg due to sorption of small molecules, in particular solvent molecules, causing an increase in the mobility of macromolecules. This topic has stimulated an abundant literature; many thermodynamic models, chiefly based on the framework set by Gibbs and Di Marzio, have been proposed^33^. Among others, Chow *et al*.^31^ proposed an explicit model for the prediction of the depression of Tg due to solvent sorption, which in the present study could give qualitative information on the plasticizing mixture to be used.

Interestingly, the preservation of both microstructure and molecule distribution of the starting microspheres enables the production of shaped microparticles that can release active VEGF over time. In particular, we demonstrate that the shaped microparticles keep a porous microstructure and VEGF/Hp/BSA distribution similar to that of the starting microspheres, whereby the release of the VEGF embedded in both types of microparticles is equivalent. By contrast, we also demonstrate that an excessive plasticization leads to closure of the particle pores and, consequently, to a higher residual VEGF activity due to a lower release. It is of our knowledge that some microfluidic^24^,^25^ and advanced non-wetting stamp based techniques^26^,^27^, as well as particle reshaping methods^29^,^34^, have been developed for the production of a broad size range of isolated, monodisperse, non-spherical microparticles. However, evidence that such microparticles can effectively release over time active form of labile proteins, such as VEGF, has not been provided so far.

The retention of the biological activity of a very labile protein, such as VEGF, lets us hypothesize that a wide variety of different drugs and proteins can be processed with the proposed method even though specific tests to assess bioactivity should be performed case by case. In addition, the retention of the microstructure suggests the possibility to tune the drug release profiles from the shaped microparticles by selecting the desired porosity of the microspheres used as starting material. Another valuable aspect is that our deformation technique allows to effectively exploit particle formulation strategies and drug encapsulation methods already developed and available for microspheres.

Finally, beside PLGA (one of the most frequently used biomaterials for microsphere production and particularly suitable even for microneedles for transdermal drug delivery)^35^,^36^, other materials employed for drug delivery and tissue engineering are likely to be plasticized at room temperature exploiting Tg depression due to solvent sorption. To this regard, we performed preliminary tests on gelatin microspheres, produced as previously reported^21^, which were deformed at room temperature employing water as a plasticizing solvent (see Supplementary Information, Fig. S4).

Since the cylindrical microparticles present a higher surface area available for drug release compared to the starting microspheres, a higher VEGF release would be expected. Although the sprout number due to the released VEGF is not significantly different between deformed and starting microspheres, it is of interest that the sprout elongation at 7 days of incubation is however higher for the deformed microspheres, presumably due to a non-monotonic mechanism of the sprouting propagation^37^. The low aspect ratio of the cylinders employed and the degradation process may account for the lack of length difference at longer incubation times.

This aspect prompts further investigations to evaluate the effects of more elongated shapes on the release profiles of the embedded biomolecules; the strength of this study, however, relies not only on the careful evaluation of the VEGF quantity entrapped within the microparticles, but also on the effects of the processing conditions on the VEGF bioactivity, which supports the possible use of our process to produce shaped polymeric microparticles preserving the effectiveness of the embedded labile biomolecules.

## Methods

### VEGF-loaded and Nile Red (NR)-loaded microspheres production

PLGA 50:50 (Resomer RG 504H) microspheres loaded with VEGF/BSA/Hp with a diameter of about 200 μm were produced with a double emulsion-solvent evaporation method, as previously reported^5^,^6^. Briefly, recombinant human VEGF was purchased from PeproTech EC Ltd (UK). VEGF and Hp quantities were both 0.1 μg per mg of microspheres. BSA was used as an aid excipient to stabilize VEGF during encapsulation and release (ratio VEGF/Hp/BSA 1:1:70 w/w/w). A VEGF/Hp/BSA solution in sterile PBS at pH 7.4 (500 μl) was poured into 2.5 ml of a PLGA solution in methylene chloride (10% w/v). The primary emulsion was generated by a high-speed homogenizer (Basic 25 equipped with a tool 6G, IKA, Germany) operating at 17,500 rpm for 1 min. Afterwards, the emulsion was added to 100 ml of 0.5% w/v aqueous PVA and stirred at 6000 rpm at room temperature (Heidolph, Germany) for 3 h to achieve solvent evaporation and subsequent microsphere hardening. Afterwards, microspheres were collected, washed 3 times with distilled water by centrifuge at 4 °C, 6000 rpm for 15 min (SL 16R, Thermo Scientific, Germany) and freeze-dried (Alpha 1-4 LSC, Christ) for 24 h (0.01 atm, −60 °C). Microspheres with a diameter ranging from 200 to 300 μm were obtained by sieving.

To evaluate the distribution of the embedded biomolecules with confocal microscopy, fluorescent microspheres containing labeled probes, i.e. BSA-Alexa-647 and Hp-Rhod 6 g, were obtained using the same procedure. To evaluate the particle microarchitecture with confocal microscopy, PLGA microspheres loaded with Nile Red (NR) as fluorophore were also produced with a Micropore® equipment (see Supplementary Information).

### Elastomeric stamp production

Three master templates were employed to produce different elastomeric stamps. The first was a Si/SU8 master, patterned with arrays of isosceles triangular prisms (L1 = 260 μm, L2 = 290 μm, H = 100 μm), while the second master was made of cyclo olefin polymer patterned with arrays of square base parallelepipeds with a pyramid at the top, i.e. micro-needles, (L = 100 μm, H = 250 μm). Differently from the first two masters, the third was produced in-house by means of a micromilling machine (Minitech Machinery, US). An array of cylindrical cavities was obtained by drilling a polymetilmethacrilate substrate with a cylindrical tip having a diameter of 200 μm for a depth of 300 μm. A second tip, having a diameter of 300 μm was employed, drilling for a depth of about 50 μm onto the previously produced cylindrical reentrances. The mold produced with the third master was provided with an array of cavities, each having a top portion and a lower portion. The top portion had a hemispherical shape with width slightly larger than the microsphere diameter, while the lower portion had cylindrical shape with an aspect ratio of 1.25. To produce the molds, a heat curable Polydimethylsiloxane (Sylgard 184, Dow Corning), 10:1 (w/w), base: curing agent, was poured onto each master template and cured while in contact with the latter at 80 °C for 2 h. Since the PMMA master had reentrant features, we used a PDMS replica, which, after sylanization, in turn served as a master template for a second PDMS replica (the final mold) that had reentrant features, or cavities. Sylanization was achieved by exposing the first PDMS replica to oxygen plasma for 1 min, rapid immersion in a solution containing 1% fluorolink, 1% acetic acid, 4% DI water and 94% isopropanol, and heating at 100 °C for 45 min.

### Determination of the plasticizing mixture

The Chow model (see Supplementary Information) for the prediction of the depression of Tg due to solvent sorption was used to determine the required range of DMC mass fraction *w* in the polymer to lower the Tg of the stock PLGA (about 48 °C) to room temperature. The Chow equation was applied to stock PLGA upon determination of the Tg and ΔCp of stock PLGA with differential scanning calorimetry (DSC). To determine the range of DMC to EtOH ratio within solution capable of effectively plasticize PLGA at room temperature while avoiding dissolution, sorption of DMC by a thin PLGA film at room temperature was monitored with a computer controlled QCM^31^,^38^ with impedance analysis (KSV QCM-Z500) using gold-coated sensors (Q sense, 5 MHz AT-cut quartz crystal). Such microbalance can also measure the dissipation (D) giving additional information on the state of the PLGA film on the quartz crystal surface. The thin film was successively exposed to DMC/EtOH solutions with a constant increase of DMC mass fractions at each injection (see Supplementary Information).

### Vaporization setup

A jacketed Drechsel bottle connected to a thermostatic bath and to a nitrogen line was used to vaporize the DMC/EtOH liquid solution. The temperature of the thermostatic bath was set to 25 °C while the nitrogen pressure was 0.1 bar. The ends of a flexible tube were connected to the gas outlet of the Drechsel bottle and to a glass funnel, respectively, so that the major base of the latter could lie onto the elastomeric stamp.

### Shaping process

PLGA microspheres were placed inside the cavities of an elastomeric mold. Each cavity had a volume similar to that of a microsphere. Next, in order to achieve room temperature plasticization, microspheres were exposed to vapors of a mixture consisting of DMC, a solvent for PLGA, and EtOH, a non-solvent for PLGA (Fig. 1). The DMC mass fractions of the tested solutions were in the range determined with the QCM measurements. Exposure time to solvent was experimentally determined at a fixed solvent mass fraction within the solution and at a fixed flow rate, i.e. about 0.145 and 20 μl/min. VEGF loaded microspheres were exposed for about 1 min while NR loaded microspheres were exposed for about 7 min. For the biological experiments, 1 mg samples of cylindrical microparticles were employed. The effectiveness of deformation was checked under a stereomicroscope before using the particles for subsequent biological experiments.

Starting and deformed microspheres were analyzed with a SEM. To evaluate their internal microstructure, particles were cut with an ultra cryomicrotome.

### Evaluation of VEGF release and bioactivity

The amount of VEGF inside the starting microspheres was quantified by a specific enzyme-linked immunosorbent assay (development Kit 900-K10, PeproTech EC Ltd, UK), while the proangiogenic activity of VEGF, as well as its effective release after deformation, was evaluated by *in vitro* sprouting angiogenesis assay, as previously reported^5^. Briefly, the proangiogenic activity of the VEGF content in 1 mg of deformed microspheres, as well as that encapsulated in 1 mg of starting microspheres, was evaluated on Human Umbilical Vein Endothelial Cells (HUVEC) and measured as sprout number and average sprout length by inspection of HUVEC cells with a confocal microscope. Furthermore, in order to evaluate the release properties of VEGF from the deformed microspheres (which is strongly dependent on their internal microstructure), the activity of VEGF released from both starting and deformed microspheres was analyzed by carrying out a proangiogenic assay after incubation at 37 °C in 1 ml of cell culture medium for 7, 14, 21, and 30 days (RT7, RT14, RT21, and RT30), respectively. The residual VEGF counterpart still entrapped within starting and deformed microspheres after incubation was also analyzed. As a comparison study, a further proangiogenic assay was carried out for microspheres deformed with the same plasticizing solution, but for different exposure times, i.e. 7 min and 1 min, and similarly incubated for 7 days (ET7).

### Statistical analysis

Quantitative data are reported as mean values ± standard deviation (SD) of three independent experiments performed in duplicate. Differences between the experimental groups have been analyzed by one-way analysis of variance (ANOVA) and p values <0.05 were considered statistically significant.

## Additional Information

**How to cite this article**: de Alteriis, R. *et al*. A method to tune the shape of protein-encapsulated polymeric microspheres. *Sci. Rep*. **5**, 12634; doi: 10.1038/srep12634 (2015).

## Supplementary Material

Supplementary Information

## References

[b1] Chen R. R. & Mooney D. J. Polymeric growth factor delivery strategies for tissue engineering. Pharm. Res. 20, 1103–1112 (2003). 12948005 10.1023/a:1025034925152

[b2] Saltzman W. M. & Olbricht W. L. Building drug delivery into tissue engineering. Nat. Rev. Drug Discov. 1, 177–186 (2002). 12120502 10.1038/nrd744

[b3] Langer R. Tissue Engineering. Mol. Ther. 1, 12–15 (2000). 10933907 10.1006/mthe.1999.0003

[b4] Van de Weert M., Jorgensen L., Horn Moeller E. & Frokjaer S. Factors of importance for a successful delivery system for proteins. Expert Opin Drug Deliv. 2, 1029–1037 (2005). 16296807 10.1517/17425247.2.6.1029

[b5] Borselli C. . Bioactivation of collagen matrices through sustained VEGF release from PLGA microspheres. J. Biomed. Mater. Res. A 92A, 94–102 (2010). 19165799 10.1002/jbm.a.32332

[b6] D’ Angelo I., Oliviero O., Ungaro F., Quaglia F. & Netti P. A. Engineering strategies to control vascular endothelial growth factor stability and levels in a collagen matrix for angiogenesis: The role of heparin sodium salt and the PLGA-based microsphere approach. Acta Biomater. 9, 7389–7398 (2013). 23523534 10.1016/j.actbio.2013.03.013

[b7] Freiberg S. & Zhu X. X. Polymer microspheres for controlled drug release. Int. J. Pharm. 282, 1–18 (2004). 15336378 10.1016/j.ijpharm.2004.04.013

[b8] Yang Y.-Y., Chung T.-S. & Ping Ng, N. Morphology, drug distribution, and *in vitro* release profiles of biodegradable polymeric microspheres containing protein fabricated by double-emulsion solvent extraction/evaporation method. Biomaterials 22, 231–241 (2001). 11197498 10.1016/s0142-9612(00)00178-2

[b9] Klose D., Siepmann F., Elkharraz K., Krenzlin S. & Siepmann J. How porosity and size affect the drug release mechanisms from PLGA-based microparticles. Int. J. Pharm. 314, 198–206 (2006). 16504431 10.1016/j.ijpharm.2005.07.031

[b10] Mitragotri S. & Lahann J. Physical approaches to biomaterial design. Nat. Mater. 8, 15–23 (2009). 19096389 10.1038/nmat2344 PMC2793340

[b11] Khademhosseini A. & Langer R. Microengineered hydrogels for tissue engineering. Biomaterials 28, 5087–5092 (2007). 17707502 10.1016/j.biomaterials.2007.07.021

[b12] Champion J. A., Katare Y. K. & Mitragotri S. Particle shape: A new design parameter for micro- and nanoscale drug delivery carriers. J. Controlled Release 121, 3–9 (2007). 10.1016/j.jconrel.2007.03.022 PMC4009069 17544538

[b13] Shim T. S., Kim S.-H. & Yang S.-M. Elaborate Design Strategies Toward Novel Microcarriers for Controlled Encapsulation and Release. Part. Part. Syst. Charact. 30, 9–45 (2013).

[b14] Kolhar P. & Mitragotri S. Polymer Microparticles Exhibit Size and Shape Dependent Accumulation around the Nucleus after Endocytosis. Adv. Funct. Mater. 22, 3759–3764 (2012).

[b15] Best J. P., Yan Y. & Caruso F. The Role of Particle Geometry and Mechanics in the Biological Domain. Adv. Healthc. Mater. 1, 35–47 (2012). 23184686 10.1002/adhm.201100012

[b16] Müller K., Fedosov D. A. & Gompper G. Margination of micro- and nano-particles in blood flow and its effect on drug delivery. Sci. Rep. 4, 4871 (2014) 10.1038/srep04871. 24786000 PMC4007071

[b17] Sullivan S. P. . Dissolving polymer microneedle patches for influenza vaccination. Nat. Med. 16, 915–920 (2010). 20639891 10.1038/nm.2182 PMC2917494

[b18] Sullivan S. P., Murthy N. & Prausnitz M. R. Minimally Invasive Protein Delivery with Rapidly Dissolving Polymer Microneedles. Adv. Mater. 20, 933–938 (2008). 23239904 10.1002/adma.200701205 PMC3519393

[b19] Prausnitz M. R. & Langer R. Transdermal drug delivery. Nat. Biotechnol. 26, 1261–1268 (2008). 18997767 10.1038/nbt.1504 PMC2700785

[b20] Nichol J. W. & Khademhosseini A. Modular tissue engineering: engineering biological tissues from the bottom up. Soft Matter 5, 1312–1319 (2009). 20179781 10.1039/b814285h PMC2826124

[b21] Imparato G., Urciuolo F., Casale C. & Netti P. A. The role of microscaffold properties in controlling the collagen assembly in 3D dermis equivalent using modular tissue engineering. Biomaterials 34, 7851–7861 (2013). 23891518 10.1016/j.biomaterials.2013.06.062

[b22] Qi H. . DNA-directed self-assembly of shape-controlled hydrogels. Nat. Commun. 4, 2275 (2013) 10.1038/ncomms3275. 24013352 PMC3768014

[b23] Tasoglu S. . Guided and magnetic self-assembly of tunable magnetoceptive gels. Nat. Commun. 5, 4702 (2014) 10.1038/ncomms5702. 25175148 PMC4153407

[b24] Hakimi N., Tsai S. S. H., Cheng C.-H. & Hwang D. K. One-Step Two-Dimensional Microfluidics-Based Synthesis of Three-Dimensional Particles. Adv. Mater. 26, 1393–1398 (2014). 24327458 10.1002/adma.201304378

[b25] Eydelnant I. A., Betty Li, B. & Wheeler A. R. Microgels on-demand. Nat. Commun. 5, 3355 (2014) 10.1038/ncomms4355. 24566526

[b26] Rolland J. P. . Direct Fabrication and Harvesting of Monodisperse, Shape-Specific Nanobiomaterials. J. Am. Chem. Soc. 127, 10096–10100 (2005). 16011375 10.1021/ja051977c

[b27] Kelly J. Y. & DeSimone J. M. Shape-Specific, Monodisperse Nano-Molding of Protein Particles. J. Am. Chem. Soc. 130, 5438–5439 (2008). 18376832 10.1021/ja8014428

[b28] Yang S. Y. . A bio-inspired swellable microneedle adhesive for mechanical interlocking with tissue. Nat. Commun. 4, 1702 (2013) 10.1038/ncomms2715. 23591869 PMC3660066

[b29] Champion J. A., Katare Y. K. & Mitragotri S. Making polymeric micro-and nanoparticles of complex shapes. Proc. Natl. Acad. Sci. 104, 11901–11904 (2007). 17620615 10.1073/pnas.0705326104 PMC1924596

[b30] Chow T. S. Molecular interpretation of the glass transition temperature of polymer-diluent systems. Macromolecules 13, 362–364 (1980).

[b31] Reviakine I., Johannsmann D. & Richter R. P. Hearing What You Cannot See and Visualizing What You Hear: Interpreting Quartz Crystal Microbalance Data from Solvated Interfaces. Anal. Chem. 83, 8838–8848 (2011). 21939220 10.1021/ac201778h

[b32] Callister W. D. Materials science and engineering: an introduction 506–507; 525–531 (John Wiley & Sons, 2007).

[b33] Mensitieri G. & Scherillo G. Environmental resistance of high performance polymeric matrices and composites. Wiley Encycl. Compos. (2012). 10.1002/9781118097298.weoc074

[b34] Sacanna S. . Shaping colloids for self-assembly. Nat. Commun. 4, 1688 (2013) 10.1038/ncomms2694. 23575692

[b35] Park J.-H., Allen M. G. & Prausnitz M. R. Biodegradable polymer microneedles: Fabrication, mechanics and transdermal drug delivery. J. Controlled Release 104, 51–66 (2005). 10.1016/j.jconrel.2005.02.002 15866334

[b36] Vecchione R. . Electro-Drawn Drug-Loaded Biodegradable Polymer Microneedles as a Viable Route to Hypodermic Injection. Adv. Funct. Mater. 24, 3515–3523 (2014).

[b37] Borselli C., Oliviero O., Battista S., Ambrosio L. & Netti P. A. Induction of directional sprouting angiogenesis by matrix gradients. J. Biomed. Mater. Res. A 80A, 297–305 (2007). 16960833 10.1002/jbm.a.30896

[b38] Battista E. . Ligand engagement on material surfaces is discriminated by cell mechanosensoring. Biomaterials 45, 72–80 (2015). 25662497 10.1016/j.biomaterials.2014.12.012

