# Supplementary material for: A method to tune the shape of protein-encapsulated polymeric microspheres
Source: Sci Rep. 2015 Jul 30;5:12634. doi: 10.1038/srep12634 (PMC4519779; doi:10.1038/srep12634)
Supplement: Supplementary Information [file srep12634-s1.pdf]

SUPPLEMENTARY INFORMATION FOR: ***"A method to tune the shape of protein-encapsulated polymeric microspheres"***

Renato de Alteriis[1,2], Raffaele Vecchione[1,2,*], Chiara Attanasio[2], Maria De Gregorio[1,2,3], Massimiliano Porzio[1], Edmondo Battista[1], and Paolo A. Netti[1,2,*]

[1]Centro di Ricerca Interdipartimentale sui Biomateriali - CRIB, Università degli studi di Napoli Federico II, Napoli, 80125, Italy

[2]Center for Advanced Biomaterials for Health Care - IIT@CRIB Istituto Italiano di Tecnologia, Napoli, 80125, Italy

[3]Dipartimento di Medicina Veterinaria e Produzioni Animali, Napoli, 80137, Italy

*nettipa@unina.it, raffaele.vecchione@iit.it

Chow model

According to the Chow model, prediction of the depression of Tg is described as follows:

$$\ln\frac{(Tg)}{(Tg0)} = \beta[(1-\theta)\ln(1-\theta) + \theta\ln\theta]$$

$$\theta = \frac{Mp}{z\,Md}\,\frac{\omega}{1-\omega}$$

$$\beta = \frac{zR}{Mp\Delta Cp}$$

where Tg0 and Tg are the glass transition temperature of the pure polymer and the glass transition temperature at a given mass fraction *w (mass of the solvent/mass of the solvent+polymer)* of solvent, respectively, Mp and Md are the molecular weights of the monomer and diluent, respectively, ΔCp is the change in specific heat of the polymer associated with its glass transition temperature, R is the gas constant, and z represents the number of macromolecules in contact with a single diluent molecule, i.e. the lattice coordination number, which can alternatively be 1 or 2.

Quartz crystal microbalance (QCM) measurements

Measurements were performed with a computer controlled quartz crystal microbalance with impedance analysis (KSV QCM-Z500) using gold-coated sensors (Q sense, 5MHz AT-cut quartz crystal).

Such microbalance can measure the dissipation (D) and different harmonics of the quartz crystal giving additional information on the state of the PLGA film on the quartz crystal surface.

Sensors were cleaned by immersion in an ammonia/hydrogen peroxide solution (2:3, v/v) at 50 °C for 5 min and then spin coated with a PLGA/DMC solution (25 mg/ml) at 2400 rpm for 120 s. Resulting PLGA film thickness was 236 nm ± 15, measured by stylus profilometry (Veeko Dektek 150).

A set of 12 DMC/EtOH solutions with a DMC mass fraction in the range 0.025-0.245 with a 0.02 increment were prepared.

Instrument temperature control was set to 25 °C and the measurement started with dry crystal. EtOH and DMC/EtOH solutions were subsequently injected in the measurement chamber every 18 min, followed by an injection of EtOH. Measurements were carried out on 3 different samples. Furthermore, the same procedure was used with an uncoated sample. Harmonics from the 1st (fundamental) to the 9th were monitored.

The weight of the polymer film was calculated taking into account film thickness, sensor measurement area (0.8 cm^2), and polymer density (1.23 g cm^{-3}). Solvent fraction in the polymer at each injection was calculated using the mass change registered by the instrument at the 3rd harmonic and taking into account the initial polymer weight. Results are summarized in Figure 2b (see Main Article), with related standard error.

The mean mass loss registered after the last EtOH injection was approximately 3.5% of the initial polymer mass. The mass change registered with the uncoated crystal was minimal, and therefore was not taken into account.

Figure S1 shows the registered dissipation change, with related standard error, at each injection.

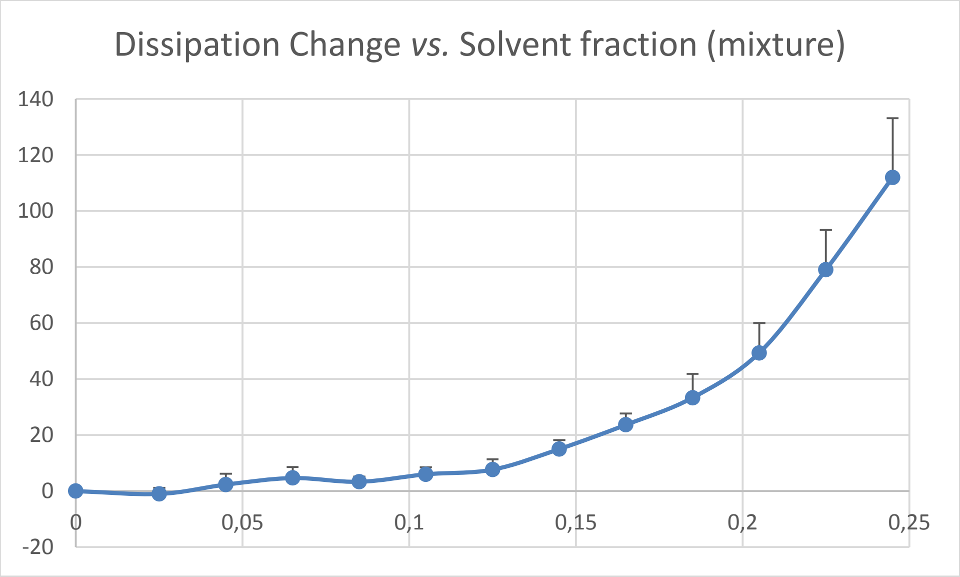

Figure S1

For each sample, dissipation change values registered for the uncoated crystal were subtracted.

SEM images of sectioned VEGF loaded microspheres and deformed microspheres

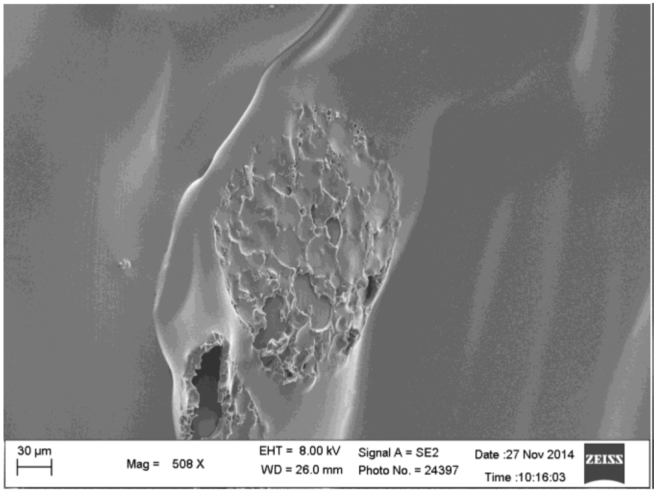

Figure S2a

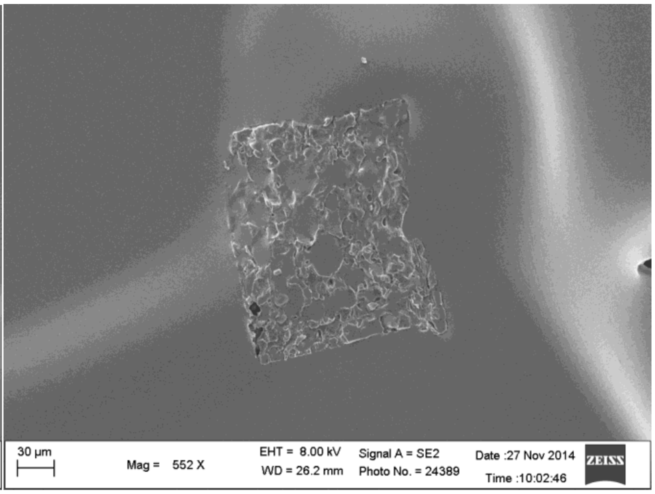

Figure S2b

Activity of the residual VEGF counterpart still entrapped in microspheres and deformed microspheres after incubation

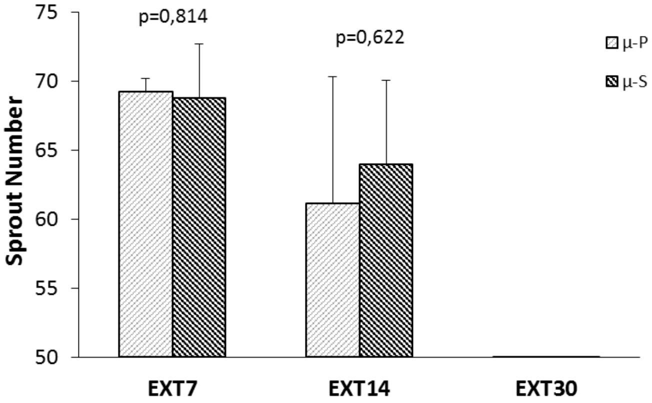

Figure S3a

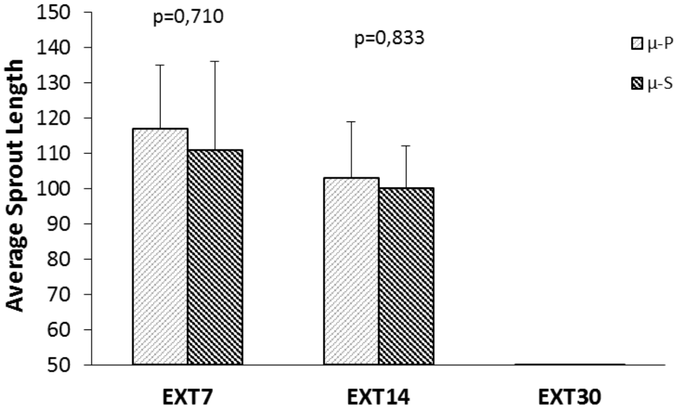

Figure S3b

Gelatin microparticles

Deformed microspheres made of gelatin (type B Sigma Aldrich Chemical Company, Bloom 225,Mw=176,654 Da) are shown in figures S4a and S4b.

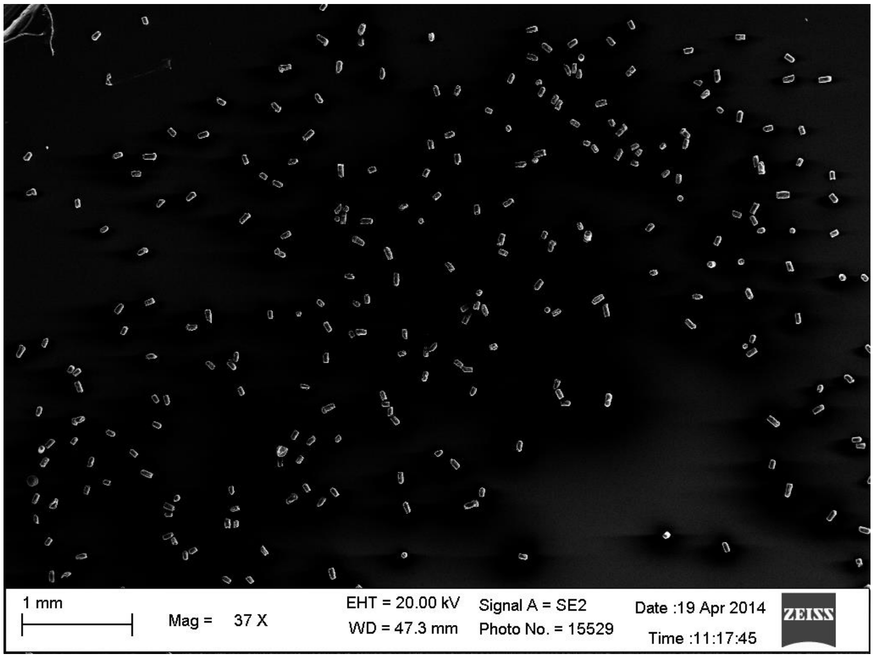

Figure S4a

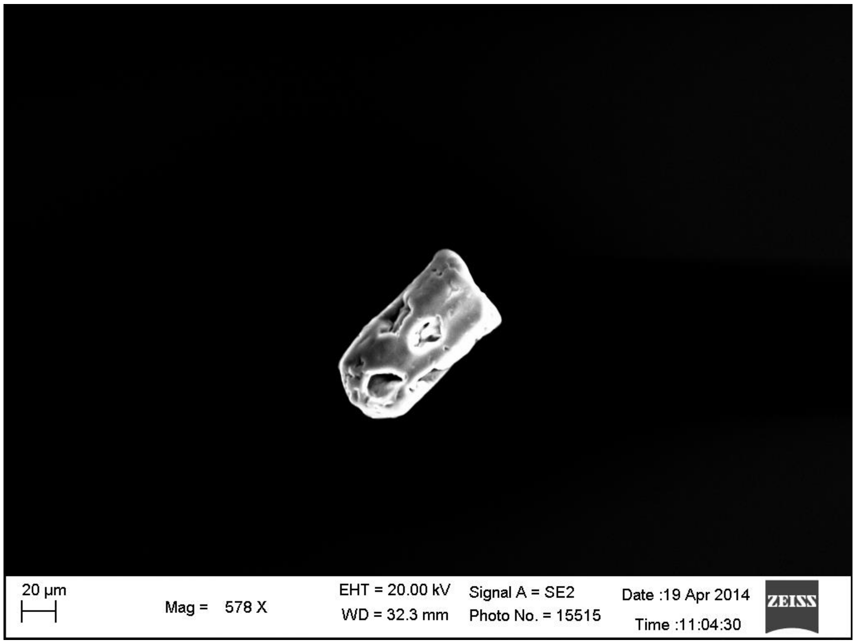

Figure S4b

NR-loaded microspheres production

NR-loaded microspheres were prepared by membrane emulsification followed by solvent evaporation. A Micropore® technologies equipment was employed. The primary emulsion was prepared adding 1 ml of DI water into 10 ml of a PLGA (Resomer RG 504H, Evonik, Germany) and Nile Red (0.1%, w/v) solution in methylene chloride (10%, w/v). W/O emulsion was generated by sonication (Branson 450, USA) setting 30% of amplitude for 30 s This emulsion was injected at 2 ml/min into the membrane equipment previously filled with 50 ml of a 1% polyvinyl alcohol (Mowiol 40-88, Sigma Aldrich) aqueous solution, where the rotational speed had been set to 276 rpm and the membrane was a hydrophilic membrane with pinhole diameters of 40 µm, and 200 µm pitch, to produce multiple emulsion W/O/W. Solvent evaporation and subsequent microsphere hardening were achieved by magnetic stirring (IKA, Germany) at room temperature. Afterwards, microspheres were collected, washed 3 times with distilled water by centrifuge at 4 °C, 6000 rpm for 15 min (SL 16R, Thermo Scientific, Germany) and freeze-dried (Alpha 1-4 LSC, Christ) for 24 h (0.01 atm, -60 °C).

VEGF-ELISA (Dosage of VEGF)

In order to determine the amount of VEGF embedded within the starting and deformed microspheres, each sample was dissolved in 1 ml of methylene chloride and the entrapped VEGF was withdrawn with 1 ml of cell culture medium (M200). VEGF in solution with the culture medium was quantified by an enzyme-linked immunosorbent assay (ELISA) according to the manufacturer's procedures (development Kit 900-K10, PeproTech EC Ltd, UK). Briefly, 100 µl of VEGF standard solution or samples appropriately diluted were added to the wells of a microplate coated with a mouse monoclonal antibody against VEGF and incubated for 2 h at room temperature. The microplates were then washed 3 times with wash buffer. After complete removal of any remaining wash buffer, the detection antibody was added to each well and incubated for 2 h at room temperature. After washing, avidin-HRP conjugate was added to each well and incubated for 30 min at room temperature. Then, the microplates were washed and 100 µl ABTS were added to each well. Following color development, the optical density (OD) was measured at λ = 405 nm with wavelength correction set at 650 nm at 5 min intervals for approximately 30 min on a microplate reader (Varian Cary 100 Scan UV/visible spectrophotometer, PelkinElmer). The linearity of the response was verified over VEGF concentration range 16–1000 pg ml–1 (r2 > 0.999). Results were expressed as encapsulation efficiency (ratio of actual to theoretical loading × 100) ± standard deviation of values collected from 3 different batches, which for the starting microspheres was 52.13 ± 3.29 and for the deformed microspheres was 45.28 ± 3.00.

Sample preparation for in vitro sprouting angiogenesis assay

The proangiogenic activity of VEGF embedded in starting and deformed microspheres, after the deformation process (ET0) and after incubation in cell culture medium at 37 °C for 7 days (ET7), was evaluated. In order to do this, two pairs of samples were prepared. Each pair consisted of 1 mg starting microspheres and 1 mg of deformed microspheres within a glass vial.
With reference to ET0, the starting and deformed microspheres were dissolved in 1 ml of methylene chloride and VEGF was withdrawn with 1 ml of cell culture medium (M200), which was used for the proangiogenic assay. With reference to ET7, after careful removal of the supernatant, the same VEGF extraction procedure was used.

The proangiogenic activity of VEGF released from starting and deformed microspheres at different times was evaluated. The activity of the residual VEGF counterpart still entrapped in microspheres and deformed microspheres was also analyzed. In order to do this, 3 pairs of samples were prepared. Each pair consisted of about 1 mg of starting microspheres and 1 mg of deformed microspheres within a glass vial. Vials were filled with 1 ml of cell culture medium (M200), and all samples were incubated at 37 °C. At predetermined time-points, i.e. 7, 14, 21, and 30 days after incubation, the supernatant (400 µl) was withdrawn from each pair and frozen at -20 °C. The supernatant at 21 and 30 days was withdrawn from the same vial. The extraction of VEGF from starting and deformed microspheres after incubation was carried out as mentioned above. The supernatant and the extracted VEGF were used for the proangiogenic assay.

Cell culture and generation of endothelial spheroids

Human Umbilical Vein Endothelial Cells (HUVECs) (Lonza) were grown in Medium 200 supplemented with LSGS kit (Life-Technologies) at 37 °C in 5% CO_2 and 100% relative humidity (RH). At early passages (II-IV) they were employed in order to generate endothelial spheroids.

After 3-4 days of culture, confluent HUVECs monolayers were trypsinized and 800 cells per spheroid were suspended in culture medium containing 0.25% (w/v) carboxymethylcellulose (Sigma), seeded into ultra-low-attachment round-bottom 96-well plates (Costar) and cultured as described to allow spheroids formation. After 24 h**,** spheroids were harvested, centrifuged at 900 rcf for 15 min, suspended in 1.2 mg/ml bovine skin collagen, transferred in 48-well plates (Falcon) and incubated. M 200 culture medium, supplemented with 2% FBS and 1% Pen Strep (10,000 U/ml penicillin G sodium, and 10,000 µg/ml streptomycin sulphate in 0.85% saline) (Gibco), were added once collagen had polymerized.

In vitro sprouting angiogenesis assay

Spheroids were divided into 4 groups of 8, and each group was used to test the bioactivity of VEGF (40 ng/ml) embedded in or released from starting and deformed microspheres. Groups were identified and treated as follows: positive control (VEGF 40 ng/ml), negative control (basal medium) and VEGF released and extracted from both starting and deformed microspheres. Positive controls were selected taking into account encapsulation efficiency and previously reported data, showing that the activity of VEGF at baseline is about 85%. Spheroids were then incubated at 37 °C, 5% CO2, and 100% RH.

After a 18-24 h culture, gels were observed by an inverted light microscope before being fixed with 4% paraformaldehyde for at least 40 min, rinsed with PBS buffer and stained with Phalloidin tetramethylrhodamine B isothiocyanate (Sigma-Aldrich) and Sytox green (Invitrogen) for actin microfilaments and cellular nuclei, respectively. Sprouting was evaluated by a Leica SP5 confocal laser scanning microscope using a HCX APO LU-V-I 10.0 X 0.30 water objective lens. Samples were excited with a 488 nm argon laser for nuclei detection, while for actin a 543nm He–Ne laser was employed. A 560–600 nm or a 505–530 nm emission was used to detect actin and nuclei, respectively. Images processing and quantitative analysis were performed by Leica LAS AF Version 2.7.3.9723 software.

Micro computed tomography images of NR loaded microspheres and deformed microspheres

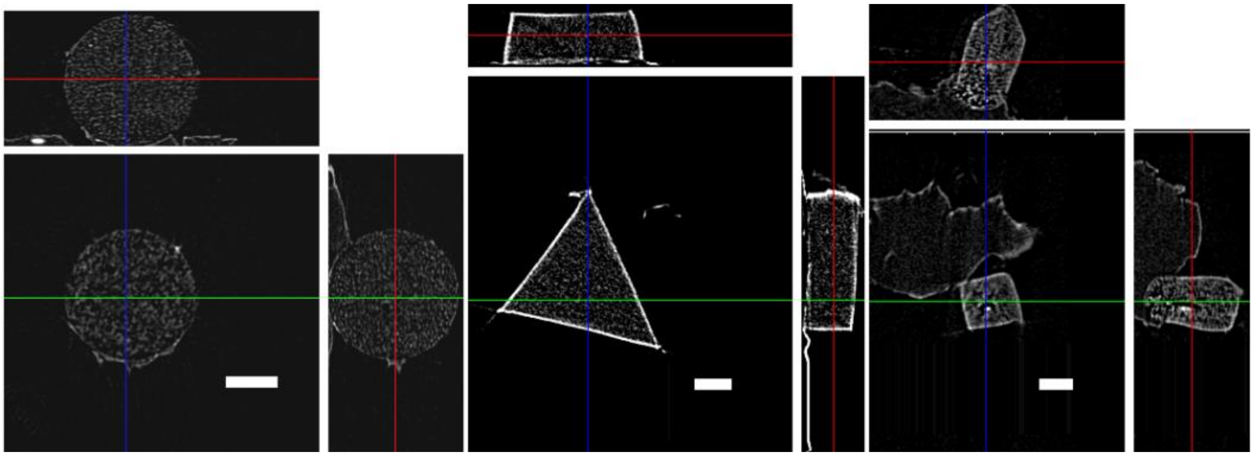

Figure S5a *Figure S5b* *Figure S5c*

DSC Thermogram

Glass transition temperature and the associate change in specific heat for stock PLGA were determined by differential scanning calorimetry (DSC) (Q20 DSC, TA Instruments).
Measurements were carried out under a constant nitrogen flow of 50 cm^3/min. Approximately 7 mg of sample were placed in a sealed aluminum pan and dynamic DSC tests were carried out over the temperature range 10–80 °C, at a heating rate of 10 °C/min, with two heating cycles.
Figure S6 shows a DSC thermogram of stock PLGA.

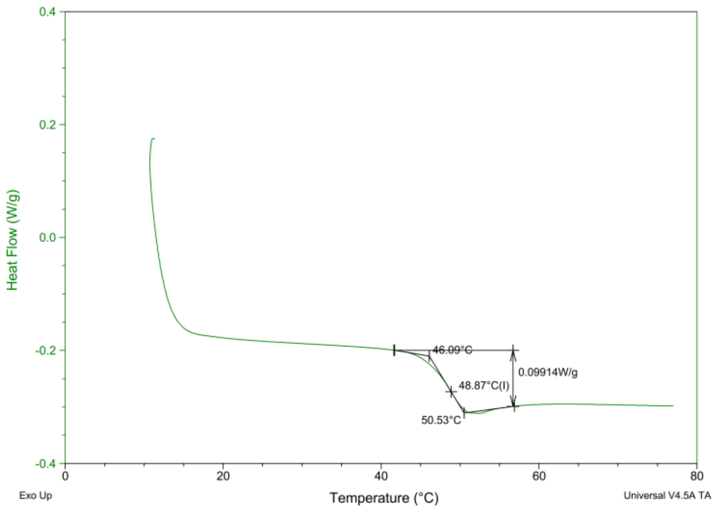

Figure S5

<u>SEM Image of PLGA cylindrical particles smaller than 10 µm</u>

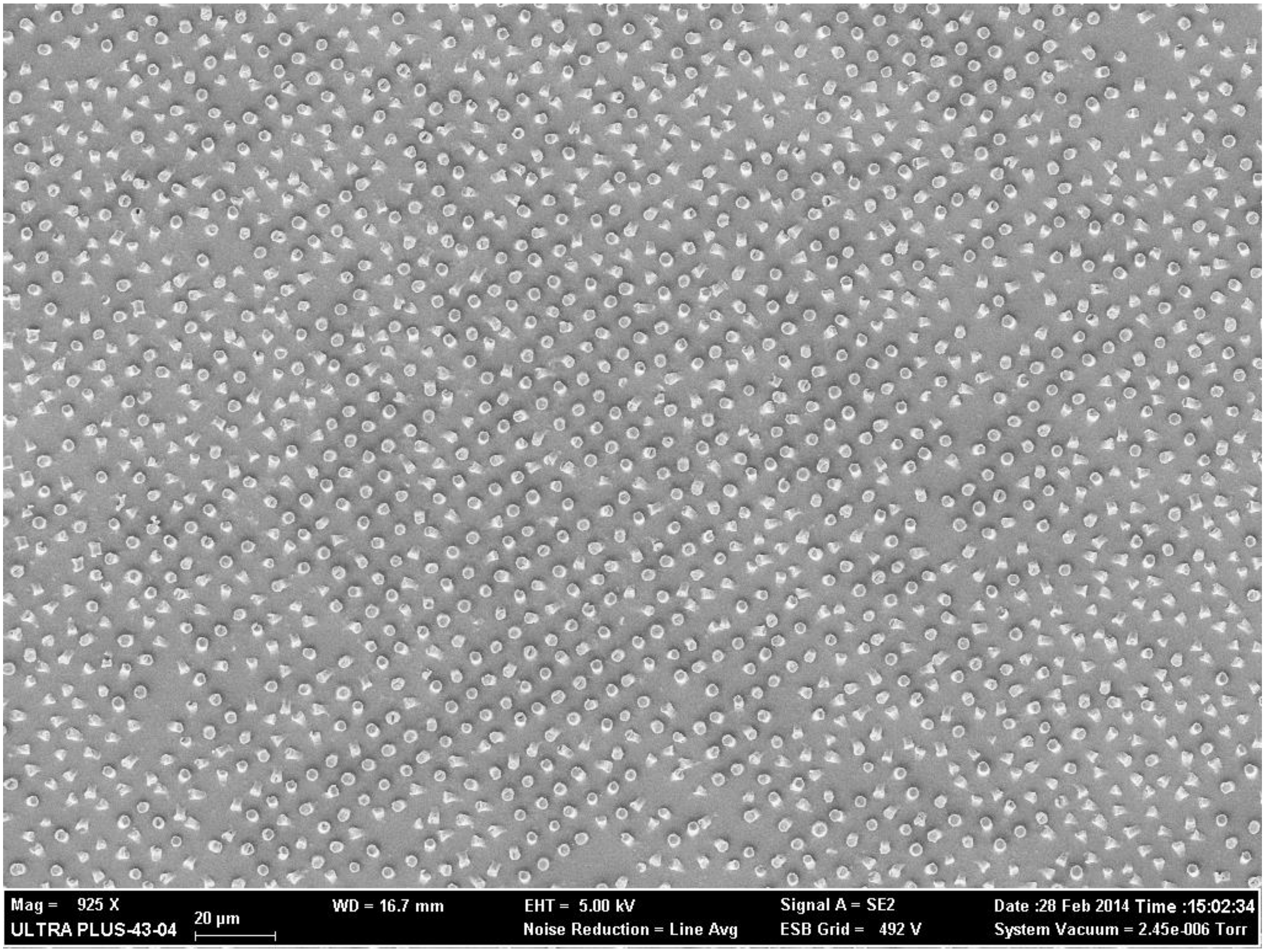

Figure S6
